# Exploration of the effects of goose TCs on GCs at different follicular stages using a co-culture model

**DOI:** 10.1042/BSR20200445

**Published:** 2020-08-07

**Authors:** Xiang Gan, Yushi Wang, Shanyan Gao, Xi Chen, Shenqiang Hu, Jiwen Wang, Jiwei Hu, Liang Li, Chunchun Han

**Affiliations:** Farm Animal Genetic Resources Exploration and Innovation Key Laboratory of Sichuan Province, Sichuan Agricultural University, Chengdu, Sichuan, China

**Keywords:** co-culture, follicle development, goose, granulosa cell, theca cell

## Abstract

Granulosa cells (GCs) play a critical role in follicular development, which cannot be separated from the assistance of theca cells (TCs). In the present study, we used a transwell system to develop three stages of goose GCs *in vitro* mono-culture and co-culture models, and we analyzed the morphology, activity, intracellular lipid content and the expression of core genes involved in *de novo* lipogenesis (DNL), steroidogenesis, proliferation and apoptosis of the GCs. In the co-culture group, the activity of all three stages of GCs showed significant (*P*<0.01) changes, and they had a strong (*P*<0.01) correlation with culture time; further, the intracellular lipid deposition of hierarchical GCs was significantly different (*P*<0.01) between the two methods. Moreover, after co-culture, in pre-hierarchical GCs, the expression of *SREBP, CYP11* and *3βHSD* was promoted (*P*<0.01). In hierarchical GCs, the expression of *ACC, SREBP, STAR, CYP11, 3βHSD* and *CCND1* was promoted at 48 h, but they were inhibited (*P*<0.05) at 96 h. In F1 GCs, the expression of *ACC, FAS, SREBP, CYP11, BCL2* and *CAS3* was inhibited (*P*<0.01). The results indicate that goose TCs had complex and time-dependent effects on the biological function of GCs at each corresponding stage, and the effects were distinct in the different stages. In addition, DNL, steroidogenesis, proliferation and apoptosis in hierarchical and F1 GCs might have some synergistic relationships in the effects of TCs on GCs. Furthermore, we speculated that TCs might play an important role in the differentiation and maturation of GCs during follicular development.

## Introduction

The granulosa cell (GC) is a part of the follicle, and it is important for follicular growth and development [1–3]. The theca cell (TC) is also a component of follicles; TCs are separated from GCs by a basement membrane, and assist GCs in regulating follicles by endocrine processes [4–7].

In mammals, GCs and TCs together constitute the steroid synthesis system of follicles, and these roles in synthesis are their main physiological functions [7,8]. Follicle synthesis includes the production of various steroids, mainly progesterone, androgen and estrogen. Progesterone is mainly synthesized in GCs, while androgens can be synthesized in TCs by using the progesterone synthesized in GC, and androgens can then be used as substrates to further synthesize estrogens in GCs [9,10]. It is clear that steroid synthesis in follicles requires the cooperation of GCs and TCs. Not only that, TCs can also secrete growth factors [11–17] to act on GC; however, in mammals, TCs have an important effect on the biological function of GCs in follicle regulation.

In contrast with mammals, the effects of TCs on GCs have specific avian characteristics [18,19]. For example, in steroidogenesis, estrogen in poultry follicles is synthesized by theca externa cells, not GCs. Moreover, the mechanism of steroid synthesis in poultry is also distinct in the different stages of follicles. In pre-hierarchical follicles, all steroids are synthesized by TC, while in hierarchical follicles, progesterone, androgen and estrogen are synthesized by GCs, theca interna and theca externa cell; that is, the pattern of steroid synthesis varies with follicle development [18,20–22].

Obviously, the effect of TCs on GCs is important for follicular development in poultry. However, there are few reports on the specific mechanisms of TCs effects on GCs at different developmental stages of follicle. Therefore, we developed a noncontact transwell co-culture system of GCs (with TCs at same stage) from goose (an important and relatively low-yielding poultry species) pre-hierarchical, hierarchical (F4–F2) and F1 follicle stages [23]; fetal bovine serum (FBS) medium was used in the models for long-term culture [24,25]. The aim was to explore the dynamic influences of TCs on GCs at different follicular development stages *in vivo*. We hope this present work will lay a theoretical foundation for the study of mechanisms of follicular development in poultry.

## Materials and methods

### Experimental animals

The healthy laying maternal line of Tianfu meat geese (Anser cygnoides, 35–45 weeks of age) was used in the present study. All geese were grown under natural conditions of light and temperature at the Experimental Farm for Waterfowl Breeding at Sichuan Agricultural University (Sichuan, China), and the geese were provided with unlimited access to feed and water. Individual laying cycles were recorded for each goose, and all geese in the same laying cycle were killed by cervical dislocation 7–9 h before oviposition. All the experiments were carried out in Sichuan Agricultural University (Sichuan, China), and all procedures in the present study were approved by the Laboratory Animal Operation Standard and Welfare Management Committee, Sichuan Agricultural University (Sichuan, China, permit No. DKY-B20141401).

### Separation of goose follicle GCs and TCs at three stages

Pre-hierarchical, hierarchical (F4–F2) and F1 follicles [23] from each ovary were dissected and washed with ice-cold sterile phosphate buffered saline (PBS, pH 7.4, Solarbio). Tweezers were used to peel away the connective tissue, and then an approximate 0.5–2.0 cm slit was cut with a surgical blade across from the stalk. The yolk and the granulosa layer flowed out. In addition, granulosa and theca tissues were washed several times with PBS to wash away the yolk separately, and the further separation and culture method for the GCs and TCs was performed as described in our previous studies [23,26].

### Mono-culture and co-culture of goose GCs

**Figure 1:**
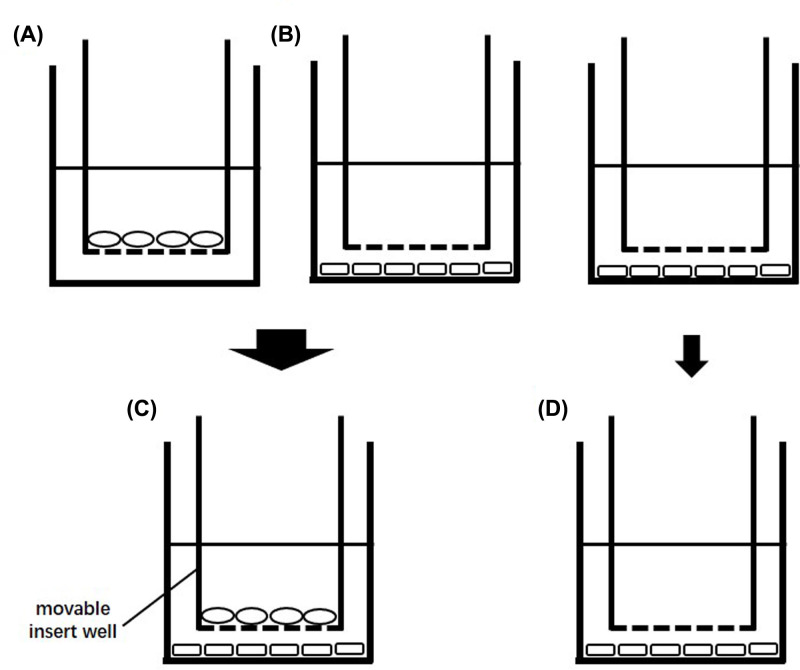
Transwell culture system and GCs and TCs culture process (**A**) TCs seeding diagram; (**B**) GCs seeding diagram; (**C**) diagram of co-culture group; (**D**) diagram of mono-culture group; The ellipses represent TCs and the rectangles represent GCs.

The three stages GCs were seeded in six-well plates (Corning) at 1.2 × 10^6^ per well with 2.5 ml of Dulbecco’s modified Eagle medium (DMEM) F-12 (HyClone) containing 10% FBS (Gibco). The corresponding stages of TCs were seeded in transwell insert wells (Corning, 6-well plates, bore diameter 0.4 μm) at 1 × 10^6^ per well in 1.5 ml of DMEM/F12 medium containing 10% FBS. Both cell types were cultured at 37°C under an atmosphere of 5% CO_2_ in humidified air. After the cells adhered (6–8 h), the culture medium was changed. In the co-culture group, two types of cells from the same follicular stage were placed in the same culture plate, and then the co-culture began (set as 0 h). For the GCs in the mono-culture group, we used a transwell insert well with no cells but containing 1.5 ml of culture medium as the control (Figure 1).

### Oil Red O strain, extraction and 3-(4, 5-Dimethylthiazol-2-yl)-2, 5-Diphenyltetrazolium Bromide Assay (MTT) assays

The activity of mono-cultured and co-cultured GCs was measured every day for 7 days by MTT (Amresco) assays, which were performed as described previously [23,26]. At 48 and 96 h, Oil red O (Solarbio) staining and extraction were performed in both mono-culture and co-culture GC groups, and the experimental method was performed according to previous literature reports [27]; to highlight the cell morphology and the effect of the Oil red O strain, we used hematoxylin (Solarbio) for restaining. Random images were captured using a microscope (Olympus) to assess cell morphology, and the OD value of Oil red O extraction in each well was monitored by an automatic enzyme immunoassay analyzer (Thermo) at 510 nm.

### Isolation of total RNA and quantitative real-time PCR

TRIzol (Invitrogen) was used to isolate total RNA from the three stages of cultured GCs at 0 h and in two methods at 48 and 96 h. The RNA was reverse-transcribed using a PrimeScript™ RT system kit for real-time PCR (TaKaRa, Japan) according to the manufacturer’s instructions. qPCRs were performed in a CFX96TM Real-Time system (Bio-Rad, CA, U.S.A.) using a SYBR PrimeScript™ real-time PCR kit (TaKaRa). qPCRs were performed in a 25 μl reaction volume that included 2.0 μl of cDNA, 12.5 μl of SYBR Premix EX Taq, 8.5 μl of sterile water, and 1.0 μl of each gene-specific primer. The raw results were repeated three times and normalized to glyceraldehyde-3-phosphate dehydrogenase (GAPDH) using the 2^−△△^Ct method [28]. Primers for the tested genes are listed in Table 1.

**Table 1:** Primer pairs for real-time quantitative PCR

Gene	Sequence (5′ to 3′)	*T*_m_ (°C)	Size (bp)
*PPARγ*	F: CCTCCTTCCCCACCCTATT	59	108
	R: CTTGTCCCCACACACACGA		
*ACC*	F: TGCCTCCGAGAACCCTAA	57	163
	R: AAGACCACTGCCACTCCA		
*FAS*	F: TGGGAGTAACACTGATGGC	57	109
	R: TCCAGGCTTGATACCACA		
*SREBP*	F: CGAGTACATCCGCTTCCTGC	60	92
	R: TGAGGGACTTGCTCTTCTGC		
*STAR*	F: AGAATCTTGACCTCTTTGACGCTG	60	87
	R: GAGACGGTGGTGGATAACGGA		
*CYP11*	F: AGGGAGAAGTTGGGTGTCTACGA	60	89
	R: CGTAGGGCTTGTTGCGGTAGT		
*3β-HSD*	F: GACCTGGGGTTTGGAATTGAG	60	170
	R: TAGGAGAAGGTGAATGGGGTGT		
*CCND1*	F: AGGAGCAGAAGTGCGAAGA	60	158
	R: TGCGGTCAGAGGAATAGTTT		
*BCL2*	F: CCTTCGTGGAGTTGTATGGCA	60	100
	R: CCACCAGAACCAAACTCAGGATA		
*CAS3*	F: CTGGTATTGAGGCAGACAGTGG	62	158
	R: CAGCACCCTACACAGAGACTGAA		
^*^GAPDH	F: GCTGATGCTCCCATGTTCGTGAT	59.6	86
	R: GTGGTGCAAGAGGCATTGCTGAC		

F, sense primers; R, antisense primers

a ^*^Housekeeping gene for data normalization

### Statistical analysis

Analyses of the differential mRNA expression data of GCs at different time points (0, 48 and 96 h) in the same cultural method and same stages were subjected to one-way ANOVA, and the means were assessed for significant differences using Tukey’s tests. Analyses of the cell activity, lipid content and mRNA expression data from the GCs in different cultural methods at different times (48 and 96 h) at the same stage were subjected to two-way ANOVA, and the means were assessed for significant differences using the Bonferroni test. The results are expressed as the mean ± SEM., and a *P*-value below 0.05 was considered statistically significant. The statistical analyses were carried out using GraphPad prism 7 [29–32].

## Results

### Morphological characteristics, growth curves and cell activity of GC at three stages in two cultural methods

**Figure 2:**
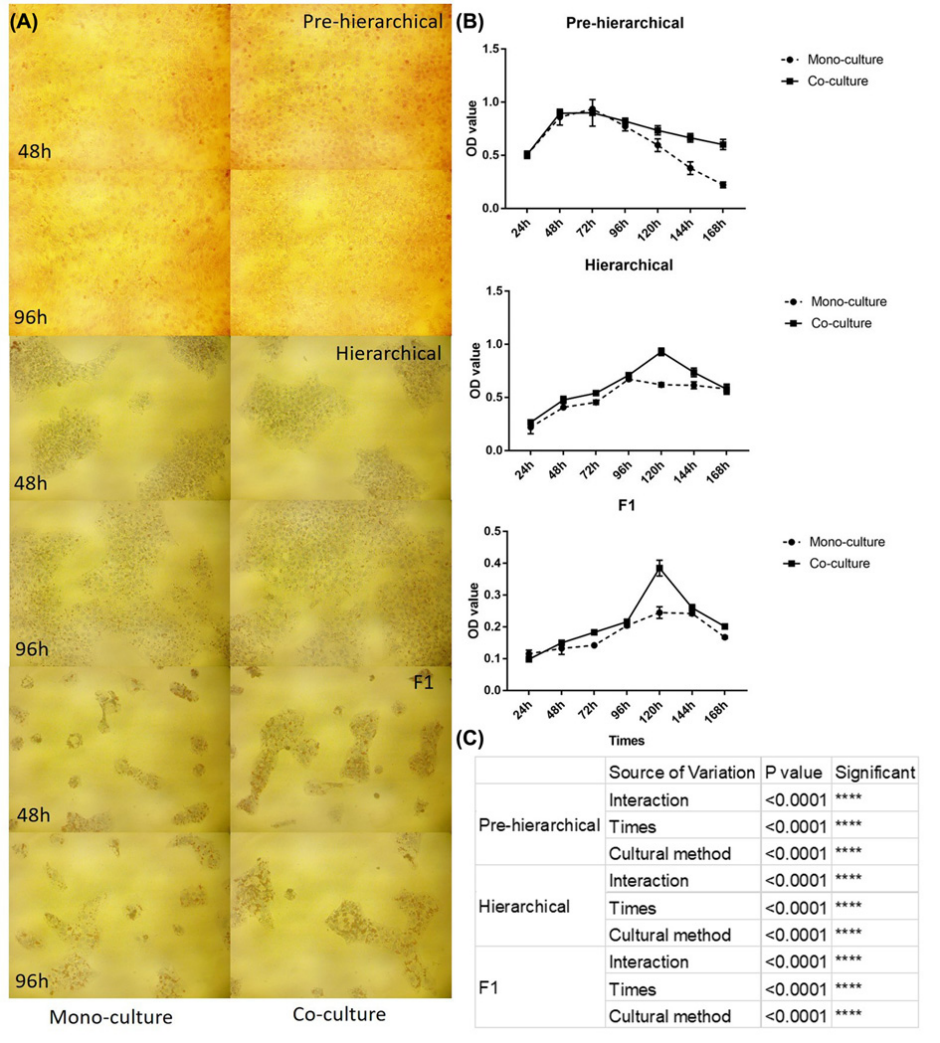
Oil red O-labeled morphology and MTT activity growth curve of GCs using the two culture methods (**A**) Photo Oil red O-labeled morphology of GCs at 48 and 96 h at three stages using a 200-fold microscope. (**B**) MTT activity growth curve of GCs for 7 days. (**C**) Two-way ANOVA for the cultural methods, culture time and GC activity.

The results showed that for pre-hierarchical GC, there was no obvious difference in cell morphology or the number of GCs in different culture methods at either 48 and 96 h (Figure 2A), which was consistent with the growth curve (Figure 2B); the divergences in growth curves occurred after 96 h. For hierarchical GCs, there was no obvious difference in the number of GCs between the two methods at 48 and 96 h (Figure 2A), which was basically consistent with the growth curve of MTT (Figure 2B). Moreover, the growth peaks between the two methods were also different; in mono-culture, the GC activity reached its growth peak at 96 h, while that of the co-culture peaked at 120 h. At the F1 stage, there was no obvious difference in cellular activity in different culture methods until 120 h. Two-way ANOVA (Figure 2C) showed that the growth activity of GCs at three stages was significantly affected by the cultural method (*P*<0.01), and the effect was closely related to the culture time (*P*<0.01).

### Oil Red O Extraction of GC

**Figure 3:**
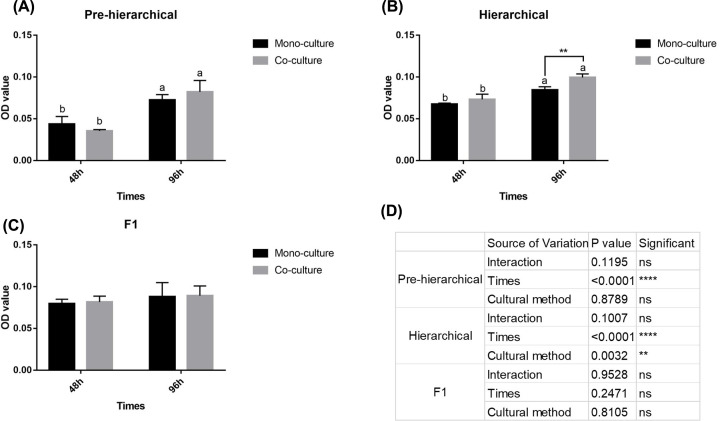
Oil red O extraction of GCs using the two culture methods (**A–C**) Oil red O extraction was performed for the two GC methods at three stages, (**D**) and two-way ANOVA was performed. The different lowercase letters (a and b) at the top of each bar represent the significant differences among lipid deposition at different time points using the same culture method and the same stages (*P*< 0.05), and the * represents the significant differences between culture methods at the same time points and stages (*P<0.05 and **P<0.01, respectively).

Oil red extraction could indicate the intracellular lipid deposition of GCs. The results showed that in pre-hierarchical and hierarchical stages, GC lipid deposition had an extremely significant (*P*<0.01) correlation with time in culture (Figure 3A,B,D), while at 96 h, the lipid deposition in GCs in hierarchical co-culture was significantly (*P*<0.01) higher than it was in mono-culture. In F1 GCs, neither culture time or method had a significant effect on lipid deposition.

### mRNA expression profiles of key *de novo* lipogenesis (DNL) genes during GC culture

**Figure 4:**
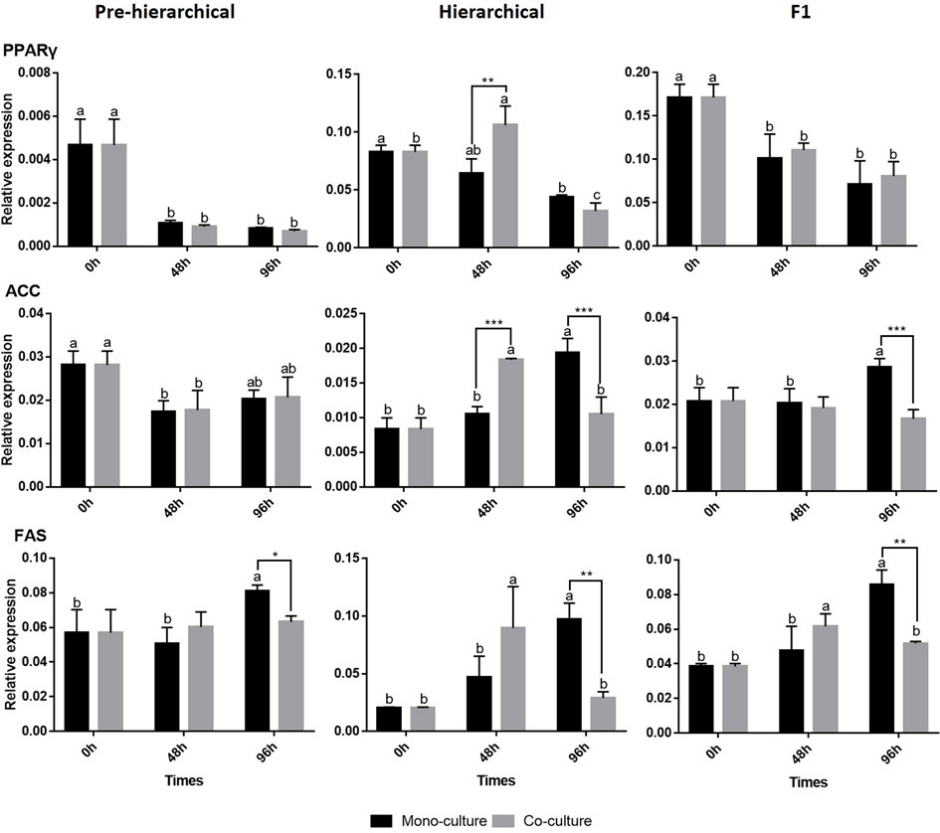
Relative expression of key DNL genes in GCs using the two culture methods The different lowercase letters (a, b, and c) at the top of each bar represent the significant differences between the gene expression at different time points using the same culture method and the same stages, and the * represents the significant differences between the culture methods at the same time points and stages(*P<0.05 and **P<0.01, respectively).

We detected the expression of key *de novo* lipogenesis (DNL) genes in GCs (Figure 4). In pre-hierarchical GCs, only *FAS* was inhibited in the 96 h co-culture method. In hierarchical GCs, co-culture promoted the expression of DNL genes at 48 h and inhibited them at 96 h. At 48 h, the expression of proliferator-activated receptors *γ* (*PPARγ*) and acetyl CoA carboxylase (*ACC*) genes in the co-culture method significantly (*P*<0.01) increased (fatty acid synthase (*FAS*) gene expression increased but not significantly), while at 96 h, *ACC* and *FAS* were significantly (*P*<0.01) decreased (*PPARγ* expression decreased but not significantly). For F1 GCs, *ACC* and *FAS* were significantly (*P*<0.01) decreased at 96 h in co-culture.

### mRNA expression profiles of key steroidogenesis pathway genes during GC culture

**Figure 5:**
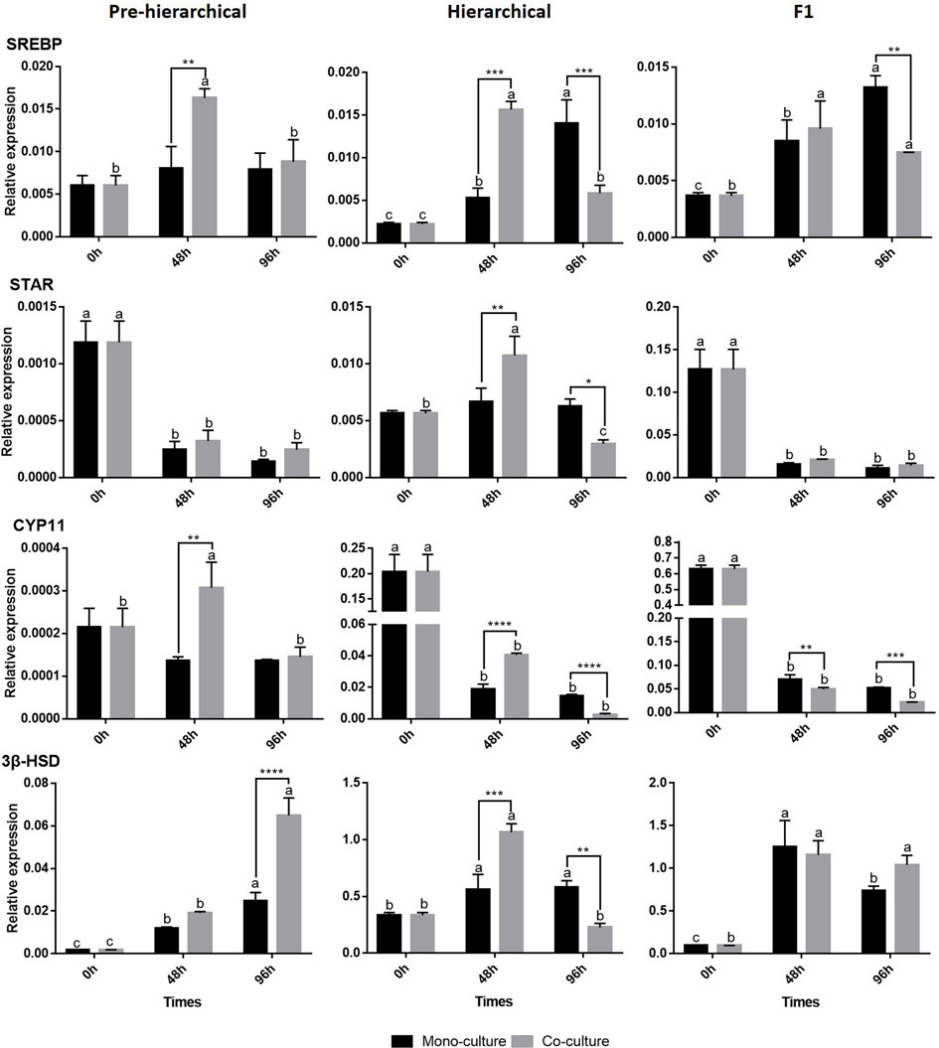
Relative expression of key steroidogenesis genes in GCs using the two culture methods The different lowercase letters (a, b, and c) at the top of each bar represent the significant differences between the gene expression at different time points using the same culture method and the same stages, and the * represents the significant differences between the culture methods at the same time points and stages(*P<0.05 and **P<0.01, respectively).

As shown in Figure 5, it was found that co-culture promoted the steroidogenesis pathway in pre-hierarchical GCs; sterol regulatory element-binding protein 1 (*SREBP*) and cholesterol side chain cleavage (*CYP11*) genes significantly increased (*P*<0.01) at 48 h, and 3β-hydroxysteroid dehydrogenase (*3βHSD*) significantly increased (*P*<0.01) at 96 h. In hierarchical GCs, co-culture had a tendency to promote and then inhibit steroidogenesis. At 48 h, co-culture significantly (*P*<0.01) promoted the expression of *SREBP*, steroidogenic acute regulatory protein (*STAR*), *CYP11* and *3βHSD*, but at 96 h, co-culture resulted in an extremely significant (*P*<0.01) inhibition of the above genes (significant (*P*<0.05) inhibition of *STAR*). In F1 GCs, co-culture significantly (*P*<0.01) inhibited both *SREBP* (96 h) and *CYP11* (48 and 96 h).

### mRNA expression profiles of key genes involved in proliferation, apoptosis and anti-apoptosis pathways in GCs

**Figure 6:**
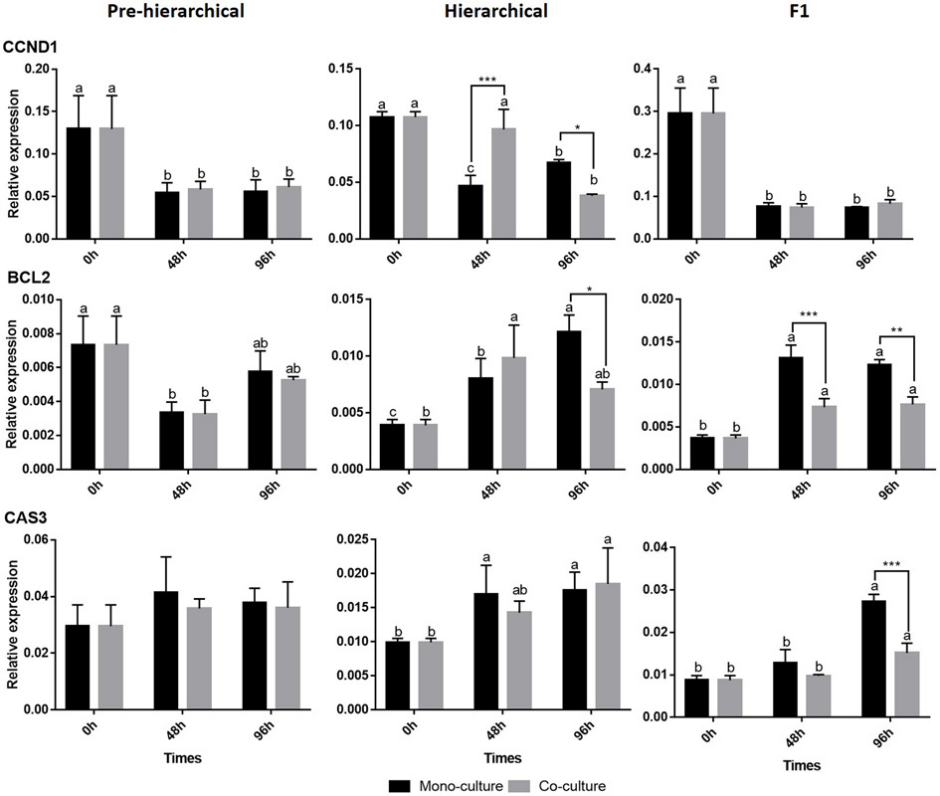
Relative expression of proliferation, apoptosis and key anti-apoptotic pathway genes in GCs using the two culture methods The different lowercase letters (a, b, and c) at the top of each bar represent the significant differences between the gene expression at different time points using the same culture method and the same stages, and the * represents the significant differences between the culture methods at the same time points and stages(*P<0.05 and **P<0.01, respectively).

As shown in Figure 6, in pre-hierarchical GCs, the culture method had no significant effect on the genes Cyclin D1 (*CCND1*), B-cell lymphoma-2 (*BCL2*) and Caspase-3 (*CAS3*). In hierarchical GCs, co-culture significantly (*P*<0.01) promoted expression of *CCND1* at 48 h and significantly inhibited *CCND1* and *BCL2* (*P*<0.05) at 96 h. In F1 GCs, co-culture significantly (*P*<0.01) inhibited *BCL2* (48 and 96 h) and *CAS3* (96 h).

## Discussion

GCs are an indispensable component of the follicular wall, and they can influence follicle recruitment, growth, selection and maturation through providing support and nutrition and through secretion of factors. TCs are adjacent to GCs, and TCs can communicate with GCs and influence their characteristics and regulation of follicles [5,24,33,34]. However, in poultry, the reports on these interactions and regulation are very limited. Therefore, in the present study, we developed a co-culture model using a transwell system to explore the effects of TCs on GCs at different developmental stages of follicle in the goose.

The activity of GCs was measured in 7-day mono-culture and co-culture groups. The results indicated that the TCs at the three stages had a certain effect on the growth activity and the trends of GCs in the corresponding stages (Figure 2A,B) [35]. In addition, as a whole (Figure 2C), TCs at all stages had a significant effect on the activity of GCs, and the effect was also significantly related to the amount of time in co-culture.

Subsequently, we detected the intracellular lipid content of GCs at three stages and found that co-culture had no significant effect on lipid deposition in F1 and pre-hierarchical GCs at 48 and 96 h (Figure 3A,B,D), which was consistent with their morphology (Figure 2A). For hierarchical follicles, co-culture time and the presence of TCs had significant effects on lipid deposition in GCs, but combined with Figure 2A, it was speculated that the increase in lipid deposition may have been partly due to the increase of GC number.

The mechanism of DNL in GCs has an important effect on follicles and is closely related to follicle proliferation, apoptosis and steroidogenesis (Figure 4) [27,36]. In hierarchical GCs, the trend of changes in DNL key genes between mono-culture and co-culture were different, suggesting that hierarchical TCs had a certain effect on DNL in GCs. Moreover, in co-culture, TCs promoted the expression of key DNL genes in GCs at 48 h, but at 96 h, their expression was inhibited. These results suggest that the mechanism of TC action on GCs in hierarchical follicles might be complex and closely related to the time in culture [37]. Co-culture had a significant inhibition of the DNL genes (*ACC* and *FAS*) in F1 GC, but it had no significant effect on DNL in pre-hierarchical GCs, indicating that TCs had different effects on GC DNL at different stages. Combined the results of Figure 3, we found that some changes in the core genes of the DNL pathway did not affect the lipid deposition of GCs, suggesting that DNL in GCs might be related to other physiological mechanisms [27].

Steroidogenesis is one of the most important functions in GCs [38–40]. The results showed that co-culture had a certain effect on the steroidogenesis mechanism of follicular GCs (Figure 5), which is consistent with the physiological pattern of TCs and GCs in poultry [41]. In pre-hierarchical follicles, co-culture had a significant effect on the core genes of steroidogenesis (*SREBP, CYP11*, and *3βHSD*) in GCs, suggesting that the effect of the TC steroid pathway on GCs existed before selection. At 96 h, *3βHSD* (a key gene in progesterone synthesis) expression in GCs in co-culture was significantly higher than it was in mono-culture. Combining these data with what is found in the literature [19,42], the pre-hierarchical follicle GCs in poultry did not have the ability to synthesize progesterone, suggesting that in pre-hierarchical follicles, TCs might induce GC development, differentiation and activation of its progesterone synthesis pathway; this conclusion is partly consistent with the GC *in vivo* developmental process [21,43]. These results emphasize the effect of TCs on GC differentiation and follicular selection. Similar to DNL in hierarchical GCs, TCs promoted the expression of key genes in the hierarchical GC steroidogenesis pathway at 48 h but inhibited them at 96 h, which confirmed that the effect of TCs on GCs was related to the time in co-culture [44]. In F1 follicles, although it has been reported that F1 GCs have a strong ability to synthesize progesterone [20–22], this activity might not be related to TC. TCs had no significant effect on the expression of *3βHSD* (Figure 5), but they could significantly inhibit the expression of *SREBP* and *CYP11*. These results indicate that co-culture had different effects on steroidogenic mechanisms at three GC stages. Such different effects of TCs on GCs at different stages were also shown in mammals [8,24,45,46], but the trends were different from those in poultry. These results suggest that TCs might play a unique role in the regulation of GC in poultry.

Proliferative and apoptotic pathways in GCs had a direct effect on follicular development (Figure 6), so we also analyzed the core genes of these processes [41,47,48]. The qPCR results of genes involved in proliferation and apoptosis in pre-hierarchical GCs cultured in the two methods were basically consistent with their growth curve (before 96 h), indicating that TCs had no significant effect on the proliferative and apoptotic pathways in pre-hierarchical GCs before 96 h. After co-culture, proliferation and apoptosis in hierarchical and F1 GCs showed significant changes, and the changes were different, suggesting that TCs had different effects on proliferation and apoptosis in GCs at different stages.

In addition, we analyzed expression patterns for GC functional pathways. In F1 GCs, the genes *BCL2, CAS3, ACC, FAS, SREBP* and *CYP11* showed similar expression trends (Figures 4–6), and they were all significantly inhibited in co-culture at 96 h (*BCL2* and *CYP11* were inhibited at both 48 and 96 h), suggesting that there might be some synergy and interaction between the anti-apoptotic, apoptotic, DNL and steroidogenesis pathways in F1 GCs. Moreover, after co-culture, the hierarchical GC gene expression of *CCND1* (Figure 6), *ACC* (Figure 4), and genes in the steroidogenesis pathway (Figure 5) also showed the same trend (increased significantly at 48 h and decreased significantly at 96 h), while the gene expression of *BCL2, PPARγ* and *FAS* also showed a similar trend (but not significant at either time point) of promotion followed by inhibition. This confirmed that TCs had a complex and time-dependent effect on hierarchical GCs [18,49] and further supported that in hierarchical and F1 follicles, there might be some synergies and interactions between proliferation, anti-apoptosis, DNL and steroidogenesis pathways when TCs are affecting GCs [27,36,50,51]. However, interestingly, the effects of TCs on the functional pathways of F1 GCs and hierarchical GCs at 96 h were similar (Figures 4–6). We speculated that the TCs of hierarchical follicles might also have the ability to induce GCs to further develop and differentiate into preovulation follicles (F1) GCs.

In summary, the present study shows for the first time that three stages of goose follicle TCs have certain effects on the cell activity, lipid metabolism, steroidogenesis, proliferation and apoptosis of GCs *in vitro*. The effects are complex, time-dependent and unique per different follicle stage. In addition, DNL, steroidogenesis, proliferation and apoptotic pathways in hierarchical and F1 GCs may have some synergistic relationship in the process of TCs regulating GCs. Furthermore, we speculate that TCs may play an important role in the differentiation and maturation of GCs during follicular development.

## Abbreviations

*3βHSD*: 3β-hydroxysteroid dehydrogenase
DNL: *de novo* lipogenesis
FBS: fetal bovine serum
GC: granulosa cell
*SREBP*: sterol regulatory element-binding protein 1
TC: theca cell
